# Tumor Immunotherapy Using Gene-Modified Human Mesenchymal Stem Cells Loaded into Synthetic Extracellular Matrix Scaffolds

**DOI:** 10.1634/stemcells.2008-0831

**Published:** 2009-03

**Authors:** Marta Compte, Ángel M Cuesta, David Sánchez-Martín, Vanesa Alonso-Camino, José Luís Vicario, Laura Sanz, Luís Álvarez-Vallina

**Affiliations:** a Molecular Immunology Unit, Hospital Universitario Puerta de HierroMadrid, Spain; b Histocompatibility Department, Centro de TransfusiónMadrid, Spain

**Keywords:** Mesenchymal stem cell, Cancer, Immunotherapy, Gene therapy

## Abstract

Mesenchymal stem cells (MSCs) are appealing as gene therapy cell vehicles given their ease of expansion and transduction. However, MSCs exhibit immunomodulatory and proangiogenic properties that may pose a risk in their use in anticancer therapy. For this reason, we looked for a strategy to confine MSCs to a determined location, compatible with a clinical application. Human MSCs genetically modified to express luciferase (MSC^luc^), seeded in a synthetic extracellular matrix (sECM) scaffold (sentinel scaffold) and injected subcutaneously in immunodeficient mice, persisted for more than 40 days, as assessed by bioluminescence imaging in vivo. MSCs modified to express a bispecific α-carcinoembryonic antigen (αCEA)/αCD3 diabody (MSC^dAb^) and seeded in an sECM scaffold (therapeutic scaffolds) supported the release of functional diabody into the bloodstream at detectable levels for at least 6 weeks after implantation. Furthermore, when therapeutic scaffolds were implanted into CEA-positive human colon cancer xenograft-bearing mice and human T lymphocytes were subsequently transferred, circulating αCEA/αCD3 diabody activated T cells and promoted tumor cell lysis. Reduction of tumor growth in MSC^dAb^-treated mice was statistically significant compared with animals that only received MSC^luc^. In summary, we report here for the first time that human MSCs genetically engineered to secrete a bispecific diabody, seeded in an sECM scaffold and implanted in a location distant from the primary tumor, induce an effective antitumor response and tumor regression.

## INTRODUCTION

Bone marrow-derived mesenchymal stem cells (MSCs) are multipotent adult stem cells of mesodermal origin localized within the bone marrow compartment. MSCs have generated a great deal of interest for their potential use in regenerative medicine and tissue engineering because of the ease of their isolation and their extensive expansion rate and differentiation potential. MSCs have been used for tissue repair, including cerebral and spinal cord injury, bone damage, myocardial ischemia/infarction, and muscular dystrophy; in the engraftment of hematopoietic stem cells (HSCs); and in the reduction of graft-versus-host disease [1, 2].

Furthermore, MSCs can be easily transduced with viral vectors to be used as cellular vehicles in gene therapy protocols [3]. The extended life span of MSCs makes them especially interesting as cell factories for the production of therapeutic proteins. In cancer therapy approaches, MSCs have been used for tumor delivery of immunostimulatory cytokines and chemokines (interferon [IFN]-α [4], IFN-β [5–8], interleukin [IL]-2 [9], IL-12 [10, 11], and CX3CL1 [12]), suicide genes (thymidine kinase [13] and cytosine deaminase [14]), growth factor antagonists (NK4) [15], and oncolytic viruses [16, 17] after systemic administration, taking advantage of their tumor-homing capacities, or by intratumoral inoculation.

However, recent evidence also suggests that they could play a role in tumor growth and metastasis [18]. The immunosuppressive [19–22] and proangiogenic properties [23] of MSCs may be responsible, at least in part, for their effects on cancer development. This implies a potential risk in the use of MSCs in cell-based anticancer therapies [24], especially when MSCs are inoculated in the vicinity of tumor cells or allowed to migrate toward them.

In fact, for strategies where cells are used as therapeutic factories, their ability to disseminate throughout the body is not required, or is even undesirable. Producer cells can be confined to scaffolds that keep cells at the implantation site, although the therapeutic protein may act at distance if secreted into circulation [25]. Subcutaneous delivery of MSCs would provide an easily accessible implant that could be retrieved once the therapeutic effect is fulfilled. It has been reported that matrix-embedded MSCs, genetically modified to produce erythropoietin (EPO) and subcutaneously inoculated in a mouse model of anemia, supported the release of the protein into the bloodstream for a sustained pharmacological effect [26, 27].

Recently, this approach was used in a breast cancer model for the delivery of IL-12 by murine MSCs embedded in a bovine collagen-based matrix [28]. Interestingly, even though plasma of mice that received the IL-12 MSC-containing subcutaneous implants showed increased levels of IL-12, there was no therapeutic effect when IL-12 MSCs were not administered in the same location as tumor cells. This requirement poses a clear limitation for the translation of this approach to a clinical setting and implies the risk of potential MSC contribution to tumor growth.

In previous works we have demonstrated the antitumoral effect of a bispecific anticarcinoembryonic antigen (α-CEA) × anti-CD3 (αCD3) diabody secreted in autocrine fashion by gene modified human primary T lymphocytes. Secreted αCEA/αCD3 diabody on tumor site induced T-cell activation and tumor growth inhibition in vivo [29, 30]. However, the short life span of activated human T lymphocytes and their inefficient transduction constitute important drawbacks for their application in cancer therapy. Alternatively, we decided to explore the potential of human MSCs as diabody producer cells in a human tumor xenograft. First, we studied the immunomodulatory properties of MSCs in our model to rule out any counterproductive effect. Conditioned medium from MSCs inhibited T-cell proliferation in vitro, and what is more, coimplantation of tumor cells and MSCs significantly promoted tumor growth in vivo. To avoid any contact with immune effectors or tumor cells, diabody-producing MSCs were embedded in a nonimmunogenic synthetic extracellular matrix (sECM) scaffold and inoculated in the ventral subcutaneous space of nude mice. Diabody was released into the bloodstream at detectable levels and inhibited the growth of human cancer cells subcutaneously inoculated in the dorsal region in the presence of systemically administered human T lymphocytes. In summary, we report here for the first time the use of human MSCs genetically engineered for the production of a bispecific diabody, seeded in an sECM scaffold and subcutaneously implanted in immunodeficient mice, to demonstrate the systemic antitumoral effect of a therapeutic antibody locally produced.

## MATERIALS AND METHODS

### Human Mesenchymal Stem Cells Isolation and Culture Conditions

Human MSCs were obtained from bone marrow samples from healthy donors after informed consent. MSCs were purified and expanded as previously described [21]. Purity of MSCs was tested by flow cytometry on a Epics XL flow cytometer (Beckman Coulter, Hialeah, FL, http://www.beckmancoulter.com) using the following monoclonal antibodies (mAbs): CD45-ECD (clone J33), CD31-fluorescein isothiocyanate (FITC) (clone 5.6E), CD34-PC5 (clone 581), CD90-PE (clone Thy1/310) (Beckman Coulter), major histocompatibility complex (MHC)-class I-FITC (clone W6/32), CD14-FITC (clone UCHM-1) (both from Sigma-Aldrich, St. Louis, http://www.sigmaaldrich.com), CD13-PE (clone L138), CD73-PE (clone AD2) (both from BD Biosciences, Erembodegen, Belgium, http://www.bdbiosciences.com), and CD105-PE (clone SN6) (eBioscience Inc., San Diego, http://www.ebioscience.com). MSCs were used for experiments between the third and fourth passages.

### Human Hematopoietic Stem Cells Isolation and Culture Conditions

Human HSCs were obtained from umbilical cord blood with informed consent and processed within 24 hours. Mononuclear cells were separated by density gradient centrifugation. CD34^+^ cell subpopulation was isolated using the MultiSort MicroBeads conjugated to anti-human CD34 mAb (clone QBEND/10) (Miltenyi Biotec, Bergisch Gladbach, Germany, http://www.miltenyibiotec.com) according to the manufacturer's instructions. Cells were stained with the following mAbs: CD38-FITC (clone HIT2) (Immunotools, Friesoythe, Germany), CD133-PE (clone AC133) (Miltenyi Biotec), CD45-ECD (clone J33), and CD34-PC5 (clone 581) (both obtained from Immunotech), and analyzed by flow cytometry. Approximately 5 × 10^4^ HSCs were cultured in 24-well plates with StemSpan SFEM Medium (Stem Cell Technologies, Vancouver, BC, Canada, http://www.stemcell.com) supplemented with a cytokine cocktail consisting of 50 ng/ml thrombopoietin, 20 ng/ml interleukin-6, 50 ng/ml stem cell factor, and 50 ng/ml flk-2/flt3 ligand (all recombinant human cytokines were from Peprotech, London, http://www.peprotech.com).

### Human Primary Peripheral Blood Lymphocytes Isolation and In Vitro Expansion

Human peripheral blood lymphocytes (PBLs) were isolated from healthy donors peripheral blood by density gradient centrifugation. Cells were expanded with Dynabeads CD3/CD28 (Dynal Biotech, Oslo, Norway, http://www.invitrogen.com/dynal) according to the manufacturer's instructions, in the presence of 100 U/ml interleukin-2 (Sigma-Aldrich). The phenotype of PBLs before and after culture was determined by flow cytometry using the following mAbs: CD45-FITC (clone B3821F4A), CD4-RD1 (clone SFC112T4D11), CD8-ECD (clone SFCI21Thy2D3), CD3PC5 (clone UCHT1) (Beckman Coulter), and CD56-PE (clone NCAM16.2) (BD Biosciences).

### Cell Lines

HEK-293 (human embryo kidney epithelia; CRL-1573) and its derivative 293T (CRL-11268) cells, HCT-116 (human colon carcinoma; CCL-247), HeLa (human cervix adenocarcinoma; CCL-2), and IMR90 (human lung fibroblast; CCL-186) cells were grown in Dulbecco's modified Eagle's medium (DMEM) supplemented with 10% heat-inactivated fetal calf serum (FCS), 2 mM l-glutamine, and penicillin/streptomycin (all from Invitrogen, Carlsbad, CA, http://www.invitrogen.com). All of these cells lines were obtained from the American Type Culture Collection (Rockville, MD, http://www.atcc.org). The HeLa^CEA^ cell line [31] was cultured in medium containing 750 μg/ml G418 (Promega, Madison, WI, http://www.promega.com).

### Lentiviral Vectors and Cell Transduction

The transfer vector pRRL-IRES-EGFP contains a cytomegalovirus promoter that drives an enhanced green fluorescent protein (EGFP) expression cassette, the vector pRRL.dAb.EGFP drives the expression of a bispecific αCEA/αCD3 diabody and EGFP, and the vector pRRL-Luc-IRES-EGFP drives the expression of luciferase and EGFP [23, 29, 30]. Lentiviral particles were produced by cotransfection of 293T cells through the calcium phosphate precipitation method [30]. Human stem cells (MSCs and HSCs), HCT-116 cells, and HEK-293 cells (1 × 10^5^) were seeded in six-well plates and infected overnight with lentivirus stocks (Lenti^EGFP^, Lenti^dAb^, or Lenti^Luc^) at a final multiplicity of infection (MOI) of 15. After 16 hours medium was replaced, and cells were cultured for 48 hours. EGFP transgene expression was monitored by flow cytometry. Conditioned media from either HSCs, MSCs, or HEK-293 cells transduced with Lenti^dAb^ (HSC^dAb^, MSC^dAb^, or 293^dAb^) were analyzed for αCEA/αCD3 diabody secretion by enzyme-linked immunosorbent assay (ELISA) as described [30].

### T Lymphocyte Proliferation Assays

Human PBLs (10^5^ cells per well) were plated in triplicate in 100 μl of complete medium in flat bottom 96-well plates, precoated (10 μg/ml) with anti-CD3 mAb (clone UCHT1; BD Biosciences). Then 100 μl of fresh medium or cell-free conditioned medium (CM) from 48-hour cultures of either MSCs (CM-MSC) or HEK-293 cells (CM-293) was added. After 72 hours cell proliferation was analyzed by the 3-(4,5-dimethylthiazol-2-yl)-2,5-diphenyltetrazolium (MTT) assay (Promega). Diabody-induced T lymphocyte proliferation assays were performed in triplicate in flat bottom 96-well plates. Human T lymphocytes (10^5^ cells per well) were cocultured with previously irradiated target cells [29] (HeLa or HeLa^CEA^) at an effector/target ratio of 5:1 in the presence of fresh medium or CM from 48-hour cultures of either lentivirally transduced HEK-293 cells or MSCs (293^EGFP^ or MSC^dAb^). After 72 hours, proliferation was analyzed by the MTT assay.

### In Vivo Bioluminescence Imaging

Mice were imaged using the ORCA-2BT high-resolution, charge-coupled device cooled digital camera (Hamamatsu Photonics France, Massy, France, http://www.hamamatsu.com) and Wasabi software (Hamamatsu Photonics). Animals were injected intraperitoneally (i.p.) with 125 mg/kg (150–200 μl) d-luciferin (Promega) 10 minutes prior to imaging. Bioluminescence imaging (BLI) was collected with a 1-minute integration time, and pseudocolor representations of light intensity were superimposed over the grayscale reference image acquired at low light (exposure time, 20 milliseconds). An average of six kinetic BLI acquisitions were collected after substrate injection to confirm a peak of photon emission. For quantification of the detected light, regions of interest were drawn, and the light emitted from each region was assessed by recording the total number of photons per second (total flux) after background subtraction.

### In Vivo Effect of MSCs on Tumor Growth

We established a xenograft model in which HCT-116 tumor cells (2 × 10^6^) were mixed, in a proportion of 4:1, with luciferase-expressing human MSCs (MSCs^Luc^) and injected subcutaneously (s.c.) into the dorsal space of 5-week-old female athymic nude mice (Hsd: athymic Nude/Nude; Harlan Ibérica, Barcelona, Spain, http://www.harlan.com). The growth kinetics of the MSC-containing HCT-116 tumors was compared with those of HCT-116 injected alone. Tumor volumes were determined at various time points using the formula width^2^ × length × 0.52. All mice were handled in accordance with the guidelines of Institutional Animal Care and Use Committee and performed in accordance with Spanish legislation.

### Tumor Cell Proliferation In Vitro

To determine the effect of CM from MSCs or normal human IMR90 fibroblasts on tumor cell proliferation in vitro, MSCs and IMR90 cells were cultured as described previously, and after 48 hours, the medium was collected and filtered. DMEM supplemented with 10% heat-inactivated FCS was used as control. HCT-116 cells (1.4 × 10^4^) were plated in triplicate in a 96-well plate and permitted to adhere, after which the culture medium was exchanged with CM containing 10% FCS. Proliferation was measured by an MTT assay at 4, 24, 48, and 72 hours.

### MSC-Seeded Scaffold Implantation

Either MSC^dAb^ or MSC^Luc^ (1 × 10^6^) were seeded in two different 90% synthetic hydrogel solutions, Extracel-X [32, 33] or Extracel-HP [34] (Glycosan BioSystems, Salt Lake City, http://www.glycosan.com), to obtain either MSC^dAb^- or MSC^Luc^-seeded scaffolds. MSC-seeded scaffolds were supplemented with 128 U/ml heparin (Sigma-Aldrich), 50 ng/ml human vascular endothelial growth factor, and 150 ng/ml human basic fibroblast growth factor (both from Peprotech) and were contralaterally implanted s.c. in the ventral area. To assess the secretion of diabody by MSC^dAb^, mice were bled by retro-orbital puncture at regular time points, and plasma concentration was determined by ELISA [30].

### Antitumoral Effect of MSC^dAb-^Seeded Scaffolds In Vivo

Luciferase-expressing HCT-116 tumor cells (HCT-116^Luc^) were inoculated s.c. into the dorsal space of nude mice as described above. Five days after tumor implantation, one group of mice received MSCs^Luc^-seeded scaffolds (sentinel scaffold) and MSC^dAb^-seeded scaffolds (therapeutic scaffold) injected s.c. in opposite flanks in the ventral area. Another group (control group) received only the MSC^Luc^-seeded scaffolds. Two days later, mice received i.v. injection (i.v.) of human T lymphocytes (2 × 10^6^). MSC-seeded scaffolds and tumors were retrieved, fixed with 10% formalin, and paraffin-embedded. Sections were stained with hematoxylin and eosin.

### Statistical Analysis

The SPSS v.14.0 software program (SPSS, Chicago, http://www.spss.com) was used for statistical analysis. The analysis of variance repeated measures model was used for compute the statistical significance of differences between groups. All *p* values were two-sided, and values of .05 or less were considered to indicate statistical significance.

### Quantitative Analyses of Tumor Vascularization

Tumors were excised at day of sacrifice and were formalin-fixed and paraffin-embedded. A histological section from each xenograft was stained with hematoxylin and eosin according to standard protocols. Sections were first scanned at low magnification (×5) to identify vascular structures. Areas of vessel density were then examined under higher magnification (×40) and counted [35]. Vascularization was quantified by enumerating the mature vessels in four randomly chosen fields and expressing the mean ± SD obtained for each condition.

## RESULTS

### Comparison of Human Hematopoietic and Mesenchymal Stem Cells Transduced with a Lentiviral Vector Encoding a Bispecific αCEA/αCD3 Diabody

The extended life span of stem cells makes them attractive as cell factories for the production of therapeutic antibodies. We wanted to compare the potential as antibody producer cells of lentivirally transduced human HSCs and MSCs. HSCs were isolated from human umbilical cord blood using human CD34 microbeads and characterized by flow cytometry analysis. More than 90% of purified HSCs exhibited the phenotype CD34^+^CD38^low^CD133^+^CD45^low^ (supporting information Fig. 1A). MSCs, obtained from human bone marrow as previously described [23], displayed a characteristic phenotype, with prominent expression of CD13, CD73, CD90, CD105, and MHC-class I, whereas CD31, CD34, and CD45 were absent (supporting information Fig. 1B).

**Figure 1:**
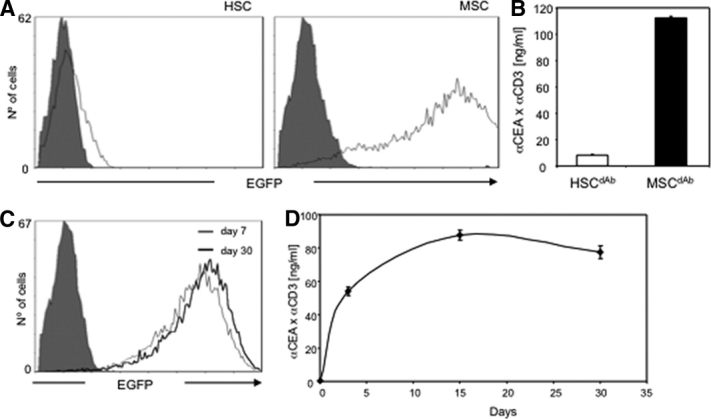
Transduction of HSCs and mesenchymal stem cells (MSCs) with dAb.EGFP-encoding lentivirus (Lenti^dAb^) at a multiplicity of infection of 10. **(A, B):** Analysis of EGFP expression **(A)** and secretion of α-CEA/αCD3 diabody into the cell culture supernatant **(B)** 72 hours after transduction. **(C, D):** Transgene expression stability in MSCs (MSC^dAb^): expression of EGFP **(C)** and secretion of α-CEA/αCD3 dAb **(D)**. Abbreviations: α-CEA, α-carcinoembryonic antigen; dAb, diabody; EGFP, enhanced green fluorescent protein; HSC, hematopoietic stem cells; MSC, mesenchymal stem cell; MSC^dAb^, mesenchymal stem cells after infection with Lenti^dAb^.

We transduced both human HSCs and MSCs with a VSV-G pseudotyped lentiviral vector encoding an αCEA/αCD3 diabody (Lenti^dAb^) [30] at an MOI of 10 to produce, respectively, HSC^dAb^ and MSC^dAb^. Flow cytometry analysis (Fig. 1A) revealed that the expression of EGFP was remarkably higher in MSCs than in HSCs. Secretion of αCEA/αCD3 diabody into the cell culture supernatant was assessed by ELISA, 72 hours after stem cell transduction (Fig. 1B). The level of functional diabody secreted by MSC^dAb^ (110 ng/ml × 10^5^ cells per 72 hours) was 10-fold higher than the observed in HSCs^dAb^ (Fig. 1B). Given that MSCs are markedly more permissive to lentiviral transduction, we decide to use MSC^dAb^ as diabody producer cells in our immunotherapeutic approach for cancer therapy.

Next, we evaluated the persistence of transgene expression by MSC^dAb^ over time post-transduction. Overall, the percentage of MSCs expressing EGFP was maintained, between 80% and 90%, for 30 days (Fig. 1C). More importantly, the secretion of functional αCEA/αCD3 diabody remained stable during this period, with levels of 81 ng/ml × 10^5^ cells per 72 hours at day 30 (Fig. 1D).

### Immunomodulatory Effect of MSCs on T-Cell Proliferation

**Figure 2:**
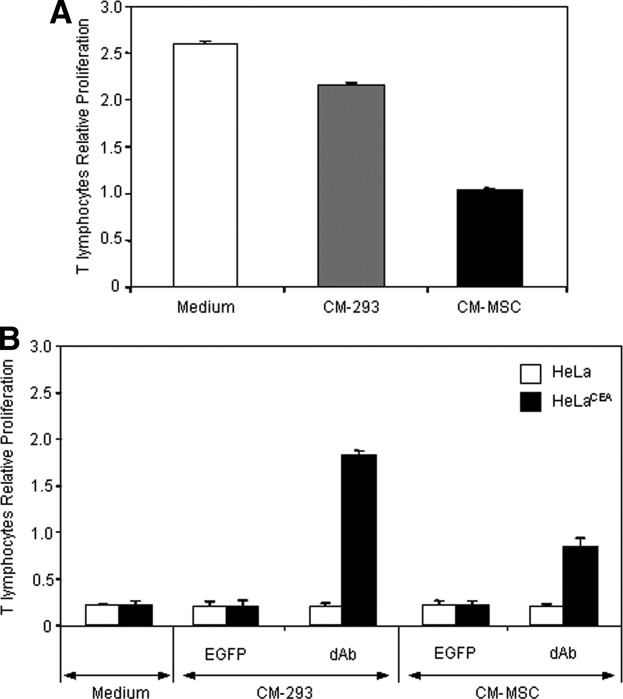
Inhibitory effect of MSCs on T lymphocyte proliferation. **(A):** CM-MSC inhibits the proliferation of human T lymphocytes following polyclonal stimuli. **(B):** CM-MSC^dAb^ inhibits the specific proliferation of T lymphocytes induced by αCEA/αCD3 diabody in presence of CEA-positive cells. Approximately 10^5^ human T lymphocytes were stimulated (effector/target ratio = 5:1) with irradiated CEA-negative (HeLa) or CEA-positive (HeLa^CEA^) target cells in the presence of medium or cell-free conditioned medium from cultures of HEK-293 or MSCs transduced with either Lenti^EGFP^ or Lenti^dAb^. As controls, effector and target cells were cultured alone (data not shown). Proliferation was analyzed after 72 hours of culture. Abbreviations: CEA, carcinoembryonic antigen; CM-293, conditioned medium from HEK-293 cells; CM-MSC, conditioned medium from mesenchymal stem cells; CM-MSC^dAb^, CM from mesenchymal stem cells transduced with Lenti^dAb^; dAb, diabody; EGFP, enhanced green fluorescent protein.

**Figure 3:**
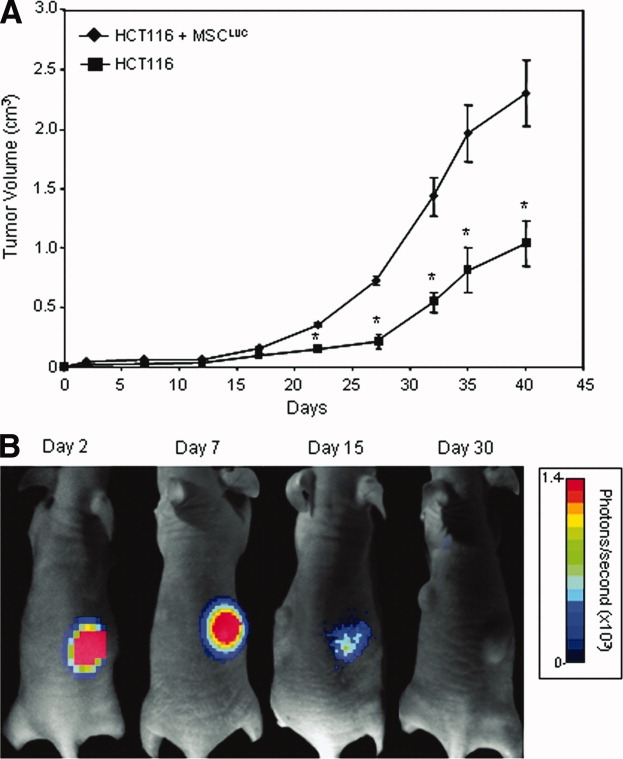
Effect of intratumoral MSCs on human colorectal carcinoma xenograft growth. **(A):** Tumor volume measurements of HCT-116 cells (2 × 10^6^) injected s.c. into nude mice (*n* = 4 mice per group) with or without 5 × 10^5^ MSC^Luc^. **(B):** The persistence of viable functional MSC^Luc^ in the tumors was assessed by bioluminescence imaging (BLI). BLI of a representative mouse is shown 2, 7, 15, and 30 days after implantation of HCT-116 and MSC^Luc^ cells. Abbreviation: MSC^LUC^, mesenchymal stem cells infected with luciferase-encoding lentivirus.

To assess the immunomodulatory effect of MSCs, human PBLs were stimulated by plastic-immobilized anti-CD3 mAb in the presence of fresh medium or cell-free CM-293 or CM-MSC. The polyclonal proliferation of T lymphocytes was roughly two times lower in the presence of CM-MSC than in the presence of either CM-293 or medium alone (Fig. 2A). As we have previously demonstrated, locally produced αCEA/αCD3 diabody is capable of acting as an efficient activating molecule for T lymphocytes in the presence of CEA-expressing tumor cells [28]. Therefore, we performed different cocultures of T lymphocytes with either CEA-positive (HeLa^CEA^) or CEA-negative (HeLa) tumor cells. CM from cultures of either HEK-293 or MSCs cells, transduced with Lenti^dAb^ or Lenti^EGFP^, was added. Diabody-induced T lymphocyte proliferation, in cocultures with HeLa^CEA^ cells, was approximately 2.5 times lower in the presence of CM-MSC^dAb^ than in the presence of CM-293^dAb^ (Fig. 2B). This difference is not attributable to a higher production of αCEA/αCD3 diabody by HEK-293 cells, given that levels were comparable in both CM as assessed by ELISA (data not shown). T lymphocytes cocultured with HeLa cells did not proliferate, independently of the source of CM (Fig. 3B). CM from EGFP-transduced cells had no effect on T-cell proliferation rate.

### Effect of MSCs on Tumor Growth

To investigate whether MSCs could exert an effect on tumor growth in vivo, we compared the growth kinetics of HCT-116 cells (a human colon carcinoma CEA-positive cell line) s.c. implanted alone (control group) or along with MSCs (ratio 4:1) in nude mice. MSCs had been previously transduced with luciferase-encoding lentiviral vector (MSCs^Luc^) to monitor in vivo their fate within tumors by BLI. Photon emission, indicating the presence of viable MSCs, was detectable until day 15 postinoculation (Fig. 3B). The subsequent loss of luciferase activity signal can be attributed to a dispersion effect due to increasing tumor volume, and not to a decrease in MSCs^Luc^ viability.

In fact, tumor growth in MSC-containing tumors (HCT-116 + MSCs^Luc^) was remarkably higher than in control mice (HCT-116 alone); the difference was statistically significant from day 17 postimplantation until the end of the experiment. At day 40, mean tumor volume in the MSCs^Luc^ group was nearly 2.5 times the tumor volume of the control group (*p* = .001) (Fig. 3A). These results strongly suggested a tumor-promoting effect of MSCs and discouraged us from taking advantage of their tumor-tropic properties. MSCs were still appealing as diabody producer cells, but special care should be taken to avoid close contact with tumor cells.

To understand the mechanism underlying the in vivo promotion of tumor growth by MSCs in our model, we studied the proliferation of tumor cells in the presence of CM-MSCs. No significant difference in cell numbers could be detected when compared with HCT-116 cells incubated with fresh medium or CM from human fibroblasts (CM-IMR90) (supporting information Fig. 2A). Then, we analyzed the effect of MSCs coimplantation on tumor angiogenesis in vivo. Quantification of microvessels in tumor sections yielded a significant increase in tumors coimplanted with MSCs (supporting information Fig. 2B). Given that no evidence of MSC malignant transformation could be detected in vivo, we can assume that the observed enhancement of tumor growth by MSCs in our model is due to increased tumor angiogenesis.

### Establishment of MSCs-Seeded Scaffolds and Systemic Release of αCEA/αCD3 Diabody

The enhancement of tumor growth and the immunomodulatory effect mediated by MSCs are important disadvantages for their use as therapeutic factories at the tumor site. To overcome these limitations, MSCs were confined to locations distant from tumor sites using injectable synthetic hydrogel scaffolds. Two different commercially available hydrogel formulations were tested for their ability to support MSC survival and engraftment: Extracel-X and Extracel-HP. At day 15 postimplantation of 10^6^ cells in nude mice, luciferase activity of MSC^Luc^ seeded in Extracel-X scaffolds was approximately twofold higher than that of MSC^Luc^ seeded in Extracel-HP (supporting information Fig. 3A). More interestingly, systemic diabody levels were three times higher in mice that received MSC^dAb^-seeded Extracel-X scaffolds (supporting information Fig. 3B). Therefore, Extracel-X was chosen for long-term studies of MSC-seeded scaffolds.

**Figure 4:**
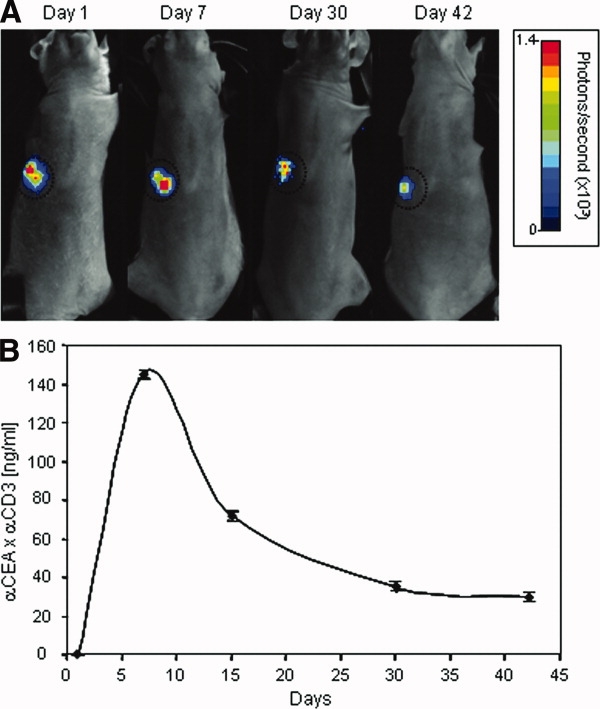
Establishment and characterization of MSCs-seeded Extracel-X scaffolds. **(A):** The persistence of viable functional MSC^Luc^ in the scaffolds was assessed by bioluminescence imaging (BLI) 1, 7, 30, and 42 days after implantation. Shown is BLI of a representative mouse (total, *n* = 3 mice per group). **(B):** Plasma concentration of αCEA/αCD3 diabody in mice implanted with MSC^dAb^-embedded scaffolds. Abbreviation: αCEA, α-carcinoembryonic antigen.

MSC^dAb^-seeded Extracel-X scaffolds (therapeutic scaffolds) were inoculated s.c. in the left ventral area of immunodeficient mice. Scaffolds containing the same amount of MSCs^Luc^ (sentinel scaffolds) were implanted contralaterally for the monitoring of MSC engraftment and persistence by BLI. As shown in Figure 4A, MSCs^Luc^-seeded scaffolds exhibited stable luciferase activity, with a slight decrease by day 42. Plasma levels of αCEA/αCD3 diabody were determined by ELISA, yielding concentrations ranging from 145 ng/ml at day 7 to 30 ng/ml 6 weeks after implantation (Fig. 4B). Histological study of MSCs^Luc^-seeded scaffolds resected 42 days after implantation revealed the presence of viable cell embedded in a dense, hematoxylin-stained matrix with no clearly differentiated architecture (data not shown).

### Antitumor Effect of MSCs^dAb^-Seeded Scaffolds in a Human Colon Carcinoma Xenograft Model

To assess the in vivo effect of αCEA/αCD3 diabody secreted by MSC^dAb^ on tumor growth, we established xenografts of the CEA-positive human colon carcinoma cell line HCT-116, previously transduced with the Lenti^Luc^ (HCT-116^Luc^), through s.c. inoculation of 10^6^ cells in the dorsal region of nude mice. Five days after tumor cell implantation, two MSC-seeded scaffolds containing, respectively, MSCs^Luc^ and MSC^dAb^ were implanted in opposite flanks in the ventral area of the same animals. A control group received only the sentinel scaffold. One week after tumor implantation the two groups of mice received an i.v. injection of 2 × 10^6^ unfractionated human PBLs. Approximately 90% of injected lymphocytes were CD3^+^: 44% CD3^+^CD4^+^ and 46% CD3^+^CD8^+^ (data not shown).

**Figure 5:**
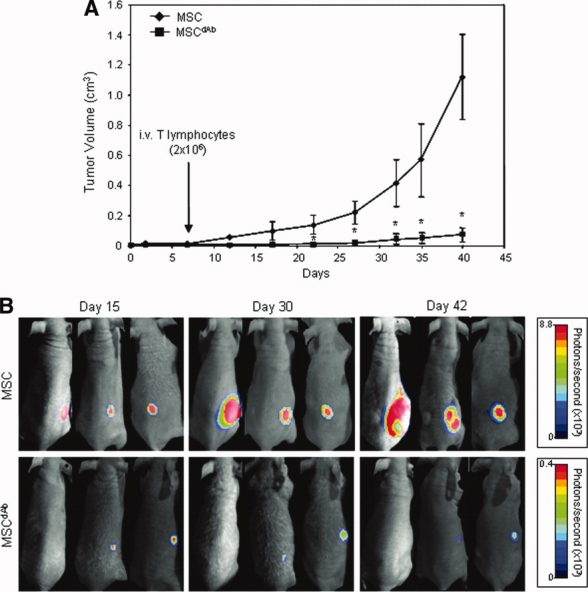
Human colorectal carcinoma xenograft growth in nude mice bearing MSC^dAb^-seeded scaffold. **(A):** Tumor volume measurements of HCT-116 cells (2 × 10^6^) infected with luciferase-encoding lentivirus (HCT-116^Luc^) injected s.c. into the dorsal skin of nude mice (*n* = 3 mice per group). Five days after tumor implantation, MSCs or MSC^dAb^ (1 × 10^6^) were seeded in a synthetic extracellular matrix Extracel-X scaffold and inoculated s.c. in the ventral area. Two days later, animals received an i.v. injection of 2 × 10^6^ human unfractionated peripheral blood lymphocytes. **(B):** Bioluminescence imaging assay for monitoring tumor growth in vivo, at days 7, 30, and 42 after tumor implantation. Abbreviations: dAb, diabody; MSC, mesenchymal stem cell.

Tumor volumes (Fig. 5A) were measured over time, and luciferase activity of HCT-116^Luc^ (dorsal) and MSCs^Luc^ (ventral) was monitored by BLI (Fig. 5B and data not shown). Mice bearing therapeutic scaffolds exhibited consistently slower tumor growth and showed a statistically significant difference in tumor volume when compared with the control group containing only sentinel scaffolds (*p* = .037 at day 40 after tumor inoculation).

## DISCUSSION

The availability of antibody genes as cloned material opens the way for alternative strategies to the passive administration of high doses of purified protein. In vivo production of therapeutic antibodies by genetically engineered cells may advantageously replace injection of purified antibodies in cancer treatment [36, 37]. The feasibility of in vivo production and systemic delivery of antibodies by different cells has been demonstrated using different techniques, such as ex vivo genetically modified autologous cells and in vivo gene transfer using viral vectors. We have previously shown that human T lymphocytes, transduced ex vivo to secrete the activating diabody αCEA/αCD3 in autocrine fashion, inhibited the growth of CEA-positive tumors [30]. However, activated T lymphocytes possess a short life span, and this implies an obvious limitation to their application in a gene therapy strategy. In contrast to terminally differentiated cell types, stem cells are endowed, at least theoretically, with a great expansion capacity. For this reason we explored the potential of HSCs, widely used in therapeutic protocols for genetic diseases, and MSCs, relatively new in the field, as cell factories for diabody production. In our hands, HSC are difficult to gene-modify, and the cytokine cocktail used for their expansion is expensive. On the contrary, MSCs are easily transduced and exhibit a unique in vitro expansion capacity using a simple medium formulation.

Several works have explored recombinant protein delivery by genetically engineered MSCs following i.v. administration or intratumoral inoculation in cancer therapy approaches [4–15]. However, MSCs have proangiogenic and immunomodulatory properties, which raise concern over the safety of their use in cancer patients [24]. MSCs are known to produce a variety of growth factors that can promote angiogenesis. Recently, we have corroborated the role of MSCs in an in vivo model of human angiogenesis, where human endothelial cells genetically modified to express luciferase were embedded in a matrix preparation (Matrigel) along with MSCs and s.c. inoculated in nude mice [23]. The presence of MSCs was essential for sustained luciferase activity, suggesting a key role of MSCs in regulating vessel maturation and functionality. More controversy exists over the effect of MSCs on tumor growth: both enhancement and inhibition, as well as no apparent effect, have been reported [24]. Intriguingly, a recent study demonstrated a role of MSCs in increasing metastasis rate, independently of the effect on primary tumor growth. In this work, MSCs accelerated the growth of MCF7/Ras tumors without affecting the kinetics of MDA-MB-231 or MDA-MB-435 tumors, but all of them displayed a marked increase in the numbers of micro- and macroscopic lung metastases [18]. We tested the effect of MSCs over human T cell-specific activation in vitro and tumor growth in vivo and found out that it could be counterproductive to let MSCs to mix with either immune effector or tumor cells. MSCs not only inhibited T-cell proliferation when cocultured with CEA-positive tumor cells in the presence of αCEA/αCD3 diabody but also significantly enhanced tumor growth in vivo. In our model, promotion of tumor growth by MSCs may be attributed to a supportive role in angiogenesis [23, 38]. Consequently, we searched for a strategy to confine MSCs to a concrete location, avoiding dissemination throughout the body.

With this aim, we took advantage of our experience with the in vivo human angiogenesis model previously described [23]. Matrigel is a murine basement membrane preparation constituted by a mixture of extracellular matrix proteins, widely used in angiogenesis studies in vitro and in vivo, but probably not best suited for MSC encapsulation in a clinical setting. We tested different hydrogel formulations, commercially available, that offer several advantages with respect to Matrigel: they are synthetic, of nonanimal origin, and potentially less immunogenic, and their composition is the same batch to batch. So we replaced Matrigel with a hydrogel sECM scaffold specifically designed for tissue engineering applications and the one that best supported MSC engraftment in vivo. This issue was addressed by BLI of MSC^luc^-seeded scaffolds and determination by ELISA of the diabody plasma levels of mice bearing MSC^dAb^-seeded scaffolds. Both luciferase activity and diabody levels in plasma were detectable 6 weeks after s.c. implantation of the respective scaffolds. Safety of implanted cells have recently been tested in a series of murine MSCs-scaffold combinations, and it was shown that the implanted cells did not spread to other organs [39].

A similar approach was described in the study by Eliopoulos et al. [26], which showed that secretion of an EPO can be prolonged by embedding the gene-modified MSCs in Matrigel before their s.c. delivery. Most interestingly, the same group recently reported the use of IL-12 producing MSCs, s.c. implanted, for breast cancer therapy [28]. In this study, Matrigel was replaced with a viscous bovine collagen-based matrix. IL-12-producing MSCs implanted in the vicinity of already present breast cancer tumor led to a significant slowing of cancer growth and to increased survival. These IL-12-secreting implants supported an increase in systemic levels of mIL-12, as concentrations were more than 10-fold higher than in the control mice. Unexpectedly, the observed therapeutic effect was not due to systemic IL-12, since IL-12-producing MSCs implanted contralaterally did not exert the antitumoral effect. This requirement for MSC implantation at the tumor site would preclude their use in a clinical setting, given that human tumors are not easily accessible in general, and MSC implants would be hardly retrievable afterward.

These results are in clear contrast with those reported here. In our study immunodeficient mice were used for T-cell transfer experiments because of the human nature of the antigens recognized by the αCEA/αCD3 diabody. In all of the experiments, an unfractionated population of human PBLs (containing CD4+ as well as CD8+ lymphocytes) was used. Diabody αCEA/αCD3 was produced and secreted into the bloodstream from a distant location, given that MSC^dAb^-seeded scaffolds were implanted in the ventral region and tumor cells in the dorsal region of nude mice. Circulating diabody was able to activate systemically transferred human T cells for the eradication of CEA-positive tumor cells, resulting in the overall reduction of tumor growth. The potential of bispecific antibodies in cancer therapy has been extensively proved in a variety of in vitro and in vivo models, and early clinical trials have shown safety of these molecules and clinical responses [40].

Nevertheless, the therapeutic potential of exogenously administered antibodies is limited by their short half-life and their poor accessibility to tumor sites. Antibody fragments exhibit rapid blood clearance and poor retention time in the target, which results in the necessity for frequent delivery of such agents. Genetically engineered cells could be the sources of sustained concentrations of soluble antibody fragments, capable of achieving long-term antitumor efficacy. In vivo production results in effective and persistent levels of antibody fragments with a syngenic glycosylation pattern. This compensates for their rapid blood clearance and could make the antibodies less immunogenic and better tolerated.

## SUMMARY

We report here for the first time the use of human MSCs genetically engineered for the production of a bispecific diabody, seeded in an sECM scaffold and s.c. implanted in immunodeficient mice, to demonstrate the systemic antitumoral effect of a therapeutic antibody locally produced in a distant location.
