# Supplementary figures and images for: Tumor Immunotherapy Using Gene-Modified Human Mesenchymal Stem Cells Loaded into Synthetic Extracellular Matrix Scaffolds
Source: Stem Cells. 2009 Mar;27(3):753–60. doi: 10.1634/stemcells.2008-0831 (PMC2729675; doi:10.1634/stemcells.2008-0831)

**A**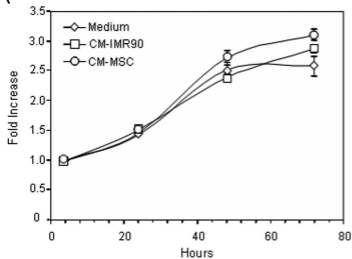**B**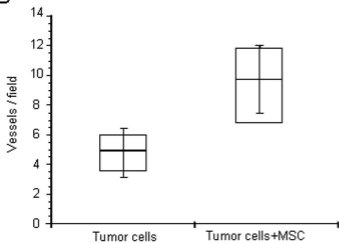

Supplementary figure 2. Compte et al.

Supplement: Supplementary file 2 [file stem0027-0753-SD2.pdf]

**A**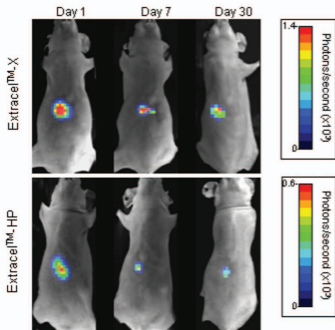**B**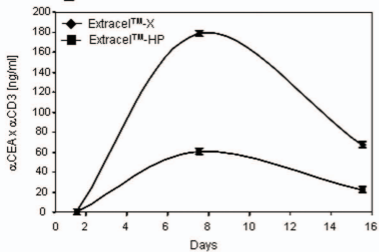

Supplementary Figure 3. Compte et al.

Supplement: Supplementary file 3 [file stem0027-0753-SD3.pdf]
